# Photosynthetic Efficiency and Anatomical Structure of Pepper Leaf (*Capsicum annuum* L.) Transplants Grown under High-Pressure Sodium (HPS) and Light-Emitting Diode (LED) Supplementary Lighting Systems

**DOI:** 10.3390/plants10101975

**Published:** 2021-09-22

**Authors:** Anna Sobczak, Marzena Sujkowska-Rybkowska, Janina Gajc-Wolska, Waldemar Kowalczyk, Wojciech Borucki, Hazem M. Kalaji, Katarzyna Kowalczyk

**Affiliations:** 1 Department of Vegetable and Medicinal Plants, Institute of Horticultural Sciences, Warsaw University of Life Sciences WULS—SGGW, Nowoursynowska 166, 02-787 Warsaw, Poland; janina_gajc_wolska@sggw.edu.pl (J.G.-W.); katarzyna_kowalczyk@sggw.edu.pl (K.K.); 2 Department of Botany, Institute of Biology, Warsaw University of Life Sciences WULS—SGGW, Nowoursynowska 159, 02-776 Warsaw, Poland; marzena_sujkowska@sggw.edu.pl (M.S.-R.); wojciech_borucki@sggw.edu.pl (W.B.); 3 Laboratory of Chemical Analysis, The National Institute of Horticultural Research, Konstytucji 3 Maja 1/3, 96-100 Skierniewice, Poland; waldemar.kowalczyk@inhort.pl; 4 Department of Plant Physiology, Institute of Biology, Warsaw University of Life Sciences WULS—SGGW, Nowoursynowska 159, 02-776 Warsaw, Poland; hazem@kalaji.pl; 5 Institute of Technology and Life Sciences, Falenty, Al. Hrabska 3, 05-090 Raszyn, Poland

**Keywords:** supplementary lighting, seedlings, leaf productivity, light quality, SPAD

## Abstract

The aim of this study was to evaluate the effects of various supplemental greenhouse lighting systems, i.e., high-pressure sodium lamps and mixtures of red and blue light-emitting diodes, on the photochemical efficiency, anatomical leaf structure, and growth of the two pepper cultivars. The intensity levels of the photosynthetically active radiation were the same for both light treatments. In this study, the relative chlorophyll content was measured. Additionally, certain parameters of chlorophyll *a* fluorescence were measured under ambient light or after dark adaptation. The obtained results showed that the application of light-emitting diodes (LEDs) as supplemental lighting positively affected the anatomical leaf characteristics and plant growth. The leaves of both pepper cultivars were thicker and had larger palisade parenchyma cells under LED supplemental lighting compared to leaves grown under high-pressure sodium (HPS) lamps. Moreover, the mesophyll cells of seedlings grown under LEDs contained more chloroplasts than those growing under HPS lighting. The chlorophyll *a* fluorescence measurements of pepper seedlings grown under LEDs showed significant increases in photosynthetic apparatus performance index (PI) values compared to plants grown under HPS lamps; however, the values for this index were higher in cv. ‘Aifos’ as compared to cv. ‘Palermo’. We recommend that supplemental lighting systems are applied with caution, as their performance appears to depend not only on the light spectrum but also on the cultivar.

## 1. Introduction

Light is one of the essential factors in a plant’s growth, development, and yield [1,2,3]. Light affects the process and intensity of photosynthesis and regulates photomorphogenesis throughout the plant development cycle; therefore, light efficiency is considered not only in terms of intensity but also in terms of wavelength [4,5,6]. This is especially important when artificial light is the only light source used in indoor farming [7]. Increasing attention is being focused on the promotion of efficient artificial light sources in lighted crops cultivated during periods when there is not enough natural light for proper plant growth and development. This applies to crops grown during winter, as well as to seedling production for the earliest crop dates [8]. Additionally, light quality can be strategically used to increase the quality and efficiency of the photosynthetic apparatus of leaves by affecting the anatomy and physiological parameters of foliage. In moderate climate zones at northern latitudes, during autumn–winter and early spring there is a deficit of the natural light required for the optimal plant growth of most plants. This is the time of year when seedlings of vegetables are usually prepared in greenhouses for the earliest cultivation dates [9]. In these periods, seedlings of vegetables destined for early cultivation are grown under covers with assimilation lighting. As the standard protocol, they are usually grown under high-pressure sodium (HPS) lamps.

As reported by Marcelis et al. [10] and Virsile, Olle, and Duchovskis [11], the levels of photosynthetically active radiation (PAR) in sodium lamps can reach approximately 1.7–1.8 µmol J^−1^. Sodium lamps have relatively high electrical efficiency as compared to the earlier generation of lamps used for light supplementation. Additionally, sodium lamps are characterized by a long working life and a broad light spectrum, making them suitable for many plant species [12]. On the other hand, HPS lamps have many disadvantages. They lack the possibility to adjust the spectral composition. They also emit a large amount of yellow light and a small amount of blue light, which causes plant stems to elongate and decreases the quality of seedlings [13]; however, despite the above-mentioned defects, these lamps were used for a long time as artificial light sources, until the appearance of LED systems [14,15].

In greenhouse crops, light-emitting diodes (LEDs) are becoming a popular source for assimilative lighting because they are much more efficient than HPS lamps and reach an average of 2.5 µmol J^−1^ or more [16]. The effectiveness and efficiency of LED lamps depend on their spectral efficiency [17]. Proper plant development depends, among other things, on the light intensity and its spectral composition [18]. LED lamps have a spectral composition adapted to the requirements of plants, high-efficiency, and a low operating temperature. They are small in size and energy-efficient, with a long life expectancy and minimal heat emissions [19]. The spectra of LED lamps are usually close to the absorption spectrum of chlorophylls, emitting red and blue light in different proportions. According to Bagdonavičienė et al. [20], blue LED light at 470 nm used as a supplement to high-pressure sodium (HPS) lamps, showing very positive effects on the photosynthesis efficiency of pepper seedlings grown during winter in a greenhouse. According to Liu et al. [2], blue light also seems to be an important factor for cherry tomato plant growth. The spectral quality also affects the anatomical structure of the plant leaves, among other factors, by influencing the arrangement of the palisade and sponge cells, which affects the photosynthetic efficiency of the plants [21,22]. According to Zheng et al. [22], in many plant species, blue light (B) and red light with blue (RB), as compared to red light (R) and white light (W), affect the growth of the palisade parenchyma, which is correlated with the quantum yield of leaf photosynthesis (φPSII). HPS and LED lighting affect the photosynthesis and yield [23]. We hypothesized that the adaptation of chloroplasts of pepper leaves to supplementary lighting provided by HPS or LED would involve changes in the distribution of chlorophylls and carotenoids. Carotenoids can absorb light energy and transfer it to the chlorophylls, which may dissipate excess light energy, acting as antioxidants [24,25]. Artificial irradiation influences the carotenoid content in leaves and fruits [23,26]. As reported by Li et al. [27], mixed R and B light alters plant photomorphogenesis and photosynthesis, mainly by affecting the leaf anatomy, stoichiometry of photosystems I and II, photosynthetic electron transport, and the expression and activity of key Calvin cycle enzymes. It was found that when pepper seedlings were illuminated with mixed RB (red–blue) light, the leaves were thicker and the photosynthetic electron transport efficiency and photosynthetic rate increased compared to plants illuminated with white light; therefore, using LED lamps with a higher proportion of blue light can increase the supplementary light efficiency. The leaf anatomy is dependent on the above-mentioned physiological and morphological changes. Proving the beneficial effects of illuminating pepper seedlings with LED lamps compared to sodium lamps may promote their use in commercial seedling production, among other outcomes. Pepper is a vegetable of great economic importance. Investigating the responses of pepper plants at the seedling stage to light from LED lamps, in terms of changes in the leaf anatomy and photochemical efficiency compared to plants grown under traditional sodium lamps, will allow the introduction of new, more energy-efficient solutions for the horticultural production of pepper plants.

The aim of the study was to evaluate the effects of supplementary lighting of pepper seedlings with HPS and LED lamps on the growth, photochemical efficiency, and anatomical structure of the leaves.

## 2. Results and Discussion

### 2.1. Plant Growth

**Figure 1:**
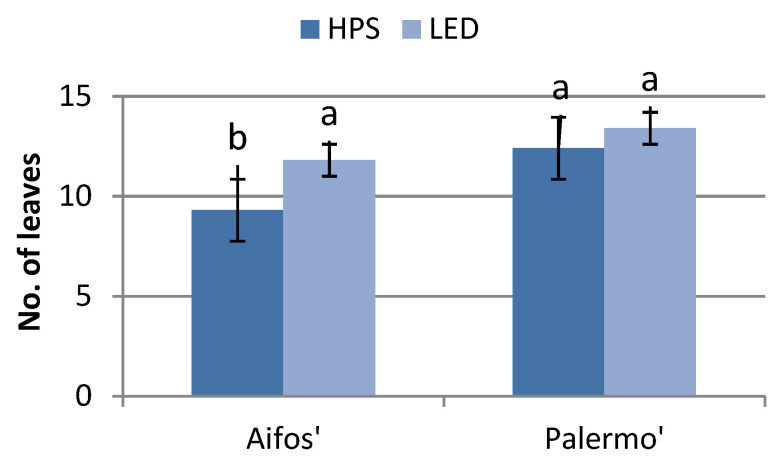
Effects of supplementary lighting with HPS and LED lamps on the numbers of leaves of pepper seedlings, depending on the cultivar. The mean values marked with the same letters do not differ significantly according to the Tukey HSD test at α = 0.05. The bars represent means ± SE (Standard Error).

**Figure 2:**
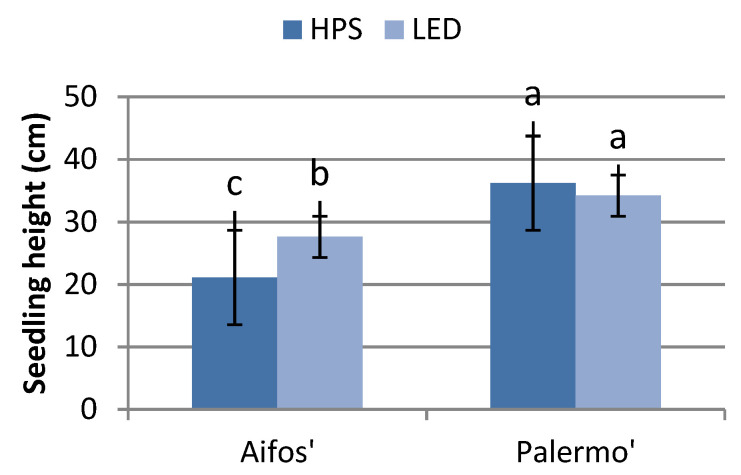
Effects of supplementary lighting with HPS and LED lamps on seedling height, depending on the cultivar. The mean values marked with the same letters do not differ significantly according to the Tukey HSD test at α = 0.05. The bars represent means ± SE (Standard Error).

Supplemental lighting affected the growth of plants and the development of leaves, depending on both the type of light source (HPS or LED) and on the cultivars. ‘Aifos’ plants growing under LED lighting conditions were significantly taller and had more leaves than those grown under HPS lighting. There was no significant effect of the lighting type on the seedling height or number of leaves in the ‘Palermo’ cultivar. On the other hand, ‘Aifos’ seedlings were shorter than the ‘Palermo’ plants, while those grown under HPS lighting also had fewer leaves (Figure 1 and Figure 2).

### 2.2. Photosynthetic Efficiency

**Figure 3:**
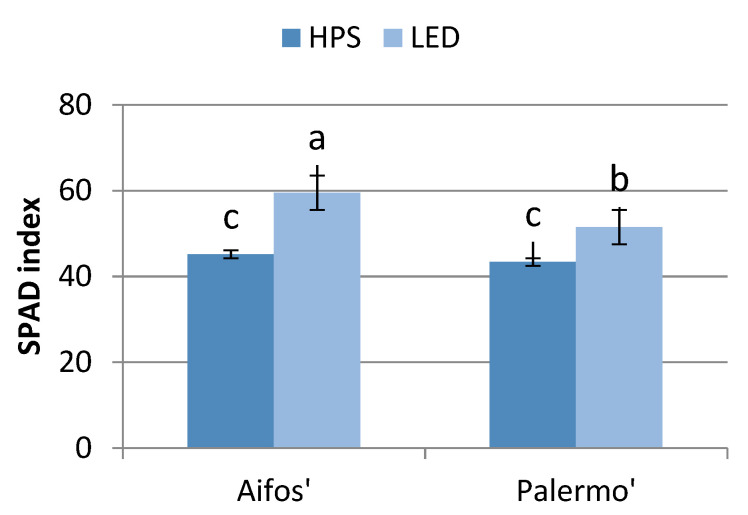
Effects of supplementary lighting with HPS and LED lamps on the chlorophyll index values in pepper leaves, depending on the cultivar (averages of four measurement dates). The mean values marked with the same letters do not differ significantly according to the Tukey HSD test at α = 0.05. The bars represent means ± SE (Standard Error).

SPAD index values are proportional to the amount of chlorophyll contained in a leaf [28]. The seedlings of both pepper cultivars grown under LED lamps were characterized by higher chlorophyll contents in the leaves than those grown under HPS lamps. The leaves of ‘Aifos’ plants were characterized by higher SPAD index values than those of ‘Palermo’ plants (Figure 3). This may have been because the spectra of LED lamps, which emit red and blue light, are more similar to the absorption spectra of chlorophyll molecules than those of HPS [29,30,31].

Inadequate lighting (the use of inappropriate supplemental lighting) can lead to decreases in photosynthesis intensity, and consequently in growth and yield. Signals of chlorophyll *a* fluorescence (ChlF) can be used to study the photosynthetic performance under varying growth conditions [23,32].

The values of chlorophyll *a* fluorescence parameters such as photosystem II (PSII), actual quantum efficiency (φPSII), and PSII maximum photochemical efficiency (Fv/Fm) values in pepper seedlings were significantly lower in pepper plants grown under LED lighting as compared to those grown under HPS (Table 1). This suggests that these parameters were not sensitive to light spectrum changes in our experiment. This could be explained by the results found in the stuides by Kalaji et al. [33,34], where it was reported that these two above-mentioned parameters are not sensitive to unfavorable growth conditions (stressors). We believe that more advanced measurement protocols should be applied in future experiments, e.g., the application of rapid light curves [35].

Generally, the performance index (PI) is a very sensitive parameter, providing quantitative information about the overall condition of plants and their vitality. Kalaji et al. [32] revealed that the PI is a very good biophysical indicator when measured under stress conditions. The use of integrative parameters such as the performance index (PI) can be more useful than a complex of specific biophysical parameters that require a deeper understanding of photochemical processes in order to interpret the data correctly. The Performance Index_Instruments_ (PI) is taken directly from the instrument and almost exactly corresponds to the Performance Index_Absorption_ (PI_Abs_). If, however, it is recalculated by selecting Fo as F 50_μs_, then PI_Abs_ = PI inst. (where Fo is the minimal chlorophyll fluorescence at time zero or 50 μs). In our work, changes of this parameter showed that the application of LEDs caused significant and positive effects on the photosynthetic efficiency levels of both tested cultivars (increases of ca. 45% and 30% for ‘Aifos’ and ‘Palermo’ cultivars, respectively) (Table 1). Better photosynthetic efficiency was also partially shown in the case of cv. ‘Aifos’, as shown by the positive trend that LEDs caused in the *Area* parameter, indicating an increase in the size of the reduced plastoquinone pool. In the same cultivar, a trend of less heat dissipation was also observed (lower DIo/RC). The above results suggest that LEDs increase the photosynthetic efficiency of plants by enhancing the light absorption, trapping, and electron transport and reducing primary acceptors of photosystem I. Additionally, as it can minimize the loss of absorbed light energy (less heat dissipation), the use of light quanta is more efficient.

Hoffmann et al. [31] showed that blue light can initiate plant responses and induce morphological and physiological changes in leaf characteristics, which also develop under high irradiance conditions; however, further investigations are required to prove the physiological mechanisms of the differences induced by different supplementary lighting systems.

### 2.3. Leaf Characteristic

**Figure 4:**
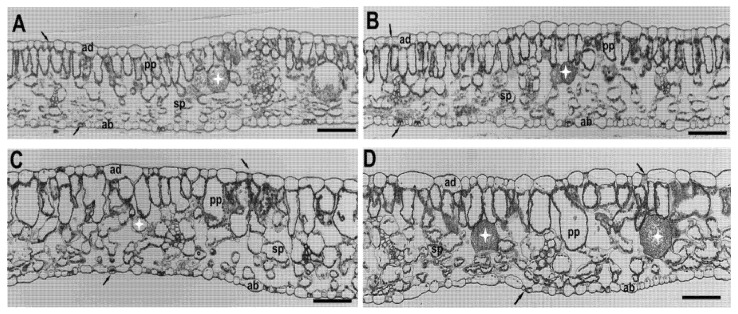
Effects of different lighting systems on the anatomies of the Capsicum leaves (cv. ‘Aifos’ or ‘Palermo’): (**A**) ‘Aifos’ HPS; (**B**) ‘Palermo’ HPS; (**C**) ‘Aifos’ LED; (**D**) ‘Palermo’ LED. Abbreviations: ad—adaxial epidermis; ab—abaxial epidermis; pp—palisade parenchyma; sp—spongy parenchyma; stomata—black arrow; calcium oxalate crystals—asterisk. Scale bar—100 µm.

The cultivars of pepper showed very similar leaf anatomies under HPS lighting, while LED lighting caused changes in leaf anatomy (Figure 4). Regardless of the cultivar and lighting, the leaves show a well-developed palisade and a spongy mesophyll. The calcium oxalate crystals were observed in vacuoles of mesophyll cells in all treatments (Figure 4).

The microscopic measurements revealed significant variations in the anatomy of the analyzed leaves (Table 2, Figure 4). The leaf thickness and cell size were significantly different between light treatments. Plants grown under LED lighting (LED) had thicker leaves than those grown under sodium lighting (HPS), with the thicknesses ranging from 264.05 μm to 276.05 μm for LED and from 193.05 μm to 195.09 μm for HPS, respectively. The changes in leaf thickness in the LED treatment were mainly due to changes in the cell size of the palisade parenchyma cells and the thickness of the spongy mesophyll, since the number of cell layers was not significantly affected by the light treatment. Similar enhanced leaf and palisade parenchyma thickness values under LED lighting conditions were observed in ornamental plants [22,32]. Reduced or no blue light results in reduced leaf thickness, while elevated blue to red levels increase the palisade and spongy mesophyll thicknesses [22]. According to Shengxin et al. [36], thicker leaves and a thicker palisade parenchyma layer result in better light absorption, and consequently higher photosynthetic yield. The LED-treated leaves showed a greater number of stomata in the adaxial epidermis compared to the HPS leaf surface (Table 2, Figure 4). A higher stomatal density, which regulates CO_2_ flux to the mesophyll, might also improve the photosynthetic efficiency [37].

### 2.4. Mesophyll Parenchyma and Chloroplast Ultrastructure of Pepper Leaves

**Figure 5:**
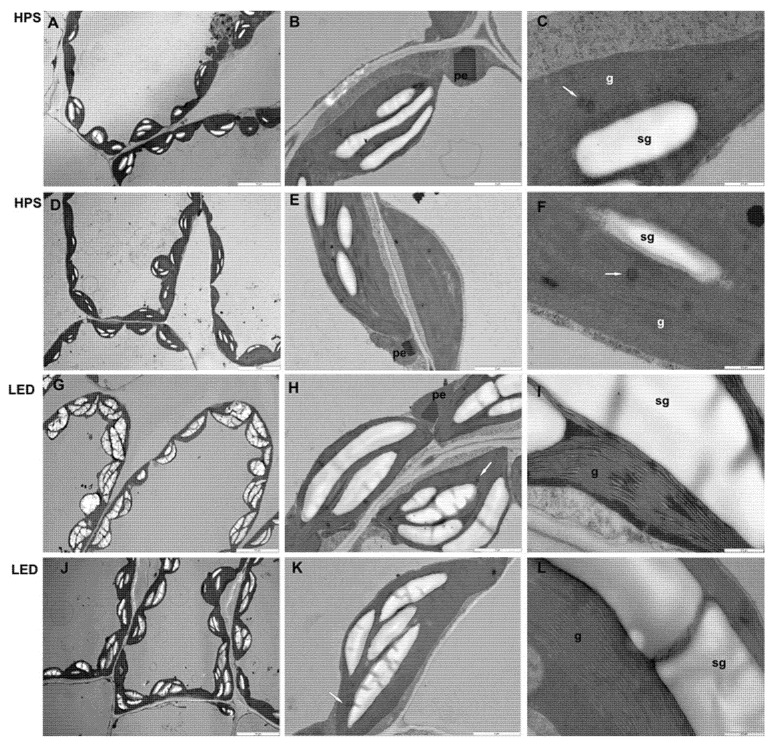
The effects of different lighting systems on the mesophyll parenchyma and chloroplast ultrastructures in pepper leaves (cv. ‘Aifos’ or ‘Palermo’): (**A**–**C**) ‘Aifos’ HPS; (**D**–**F**) ‘Palermo’ HPS; (**G**–**I**) ‘Aifos’ LED; (**J**–**L**) ‘Palermo’ LED. Abbreviations: pe—peroxisome; white arrow—plastoglobule; sg—starch granule; g—granum; HPS—sodium lamp; LED—light-emitting lamp. (**A**,**B**,**D**,**E**,**G**,**H**,**J**,**K**) Scale bar—2 µm. (**C**,**F**,**I**,**L**) Scale bar—0.5 µm.

The mesophyll parenchyma of the pepper plants showed significant differences with different light treatments, although significant differences were not observed between the cultivars (Figure 5). Mesophyll cells under LED lighting contained larger chloroplasts and more chloroplasts rich in starch grains than those growing under sodium lamps. The shape of chloroplasts grown under LED lighting was similar to that under HPS light; however, the chloroplasts in plants grown under LED lighting were enlarged and possessed thicker grana than those grown under sodium light treatment. Moreover, in chloroplasts in LED-grown leaves, a few small plastoglobules were observed. In this study, the denser arrangement of thylakoids in plants grown under LED lighting may have contributed to the increase in chlorophyll *a* content, since this molecule is crucial for grana formation and stabilization [38,39,40].

As the chlorophyll content directly influences the photosynthetic potential [41], the increased number of chloroplasts with thicker grana in the mesophyll of LED-grown leaves may have contributed to the higher photosynthetic performance of this treatment. This is consistent with the results of a study on cucumber leaves and cotton leaves grown under blue LED lighting, which also showed a greater number of chloroplasts and increased integrity of the chloroplast ultrastructure, with a clearly visible lamellar structure [42,43]. During the LED response, modifications of the thylakoid membrane system in pepper plants were accompanied by starch accumulation, which may indicate a low level of stress in those plants or indicate higher photosynthetic activity. The increased daily starch turnover resulting from induced activity of α- and β-amylases (the main starch-degrading enzymes) was unequivocally related to stress conditions [44]. Additionally, the appearance of small plastoglobules in chloroplasts under LED lighting might indicate a low level of oxidative stress, since an increased number of these structures in chloroplasts is connected with reactive oxygen species accumulation [45].

### 2.5. Fluorescence of Chlorophyll and Carotenoids

**Figure 6:**
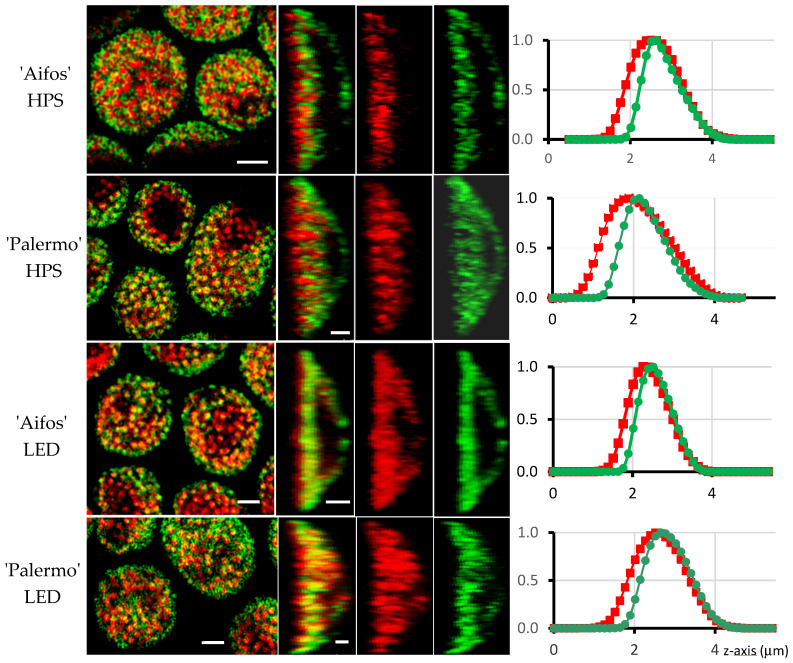
Fluorescence of chlorophyll and carotenoids (false red and green colours, respectively) in chloroplasts of pepper leaves (cv. ‘Aifos’ or ‘Palermo’). Sun light was supplemented with sodium lamps (HPS) or diode (LED) actinic light. First (left) column: fluorescence of chlorophyll and carotenoids (merged images) in single median optical sections (x:y mode of scanning) through groups of chloroplasts. Notice that grana structures are represented by red discs. The second column represents median optical sections along *z*-axis of chloroplasts: fluorescence of chlorophyll and carotenoids—merged images. Last two columns: fluorescence of chlorophyll or carotenoids is shown separately. Line graphs show changes in fluorescence intensity (*y*-axis, normalized) of chlorophyll (red line) and carotenoids (green line) along *z*-axis (µm) of chloroplasts starting from their bottom side (value 0) to the top (value 4) of the organelles. Notice that fluorescence of chlorophyll always exceeds that of carotenoids when starting from the bottom of chloroplasts to their top. Abbreviations: star—starch granule. Scale bars—2 µm.

Images in the first column (Figure 6) show the distribution of chlorophyll (false red color) and carotenoid (false green color) fluorescence signals in the upper section of the chloroplasts. The red spots probably represent granum structures [41]. Independently of the cultivar and source of additional light, chlorophyll and carotenoid signals occur in the grana, as well as between them. It seems that under LED lighting conditions, the fluorescent signals for chlorophyll and carotenoids are stronger than under HPS sodium lighting conditions, as can be seen by comparing the images in the second column.

The fluorescence of chlorophyll always exceeds that of the carotenoids when starting from the ‘bottom’ of chloroplasts and moving to the ‘top’ (Figure 6, line graphs). The bottom of the chloroplast is defined as its site (often flat), which is directed toward the cell wall, while the top (usually convex shape) is directed toward the central vacuole (Figure 6). Compared to LED lighting, the increase in chlorophyll fluorescence was significantly greater than for carotenoids under HPS lighting conditions. Several studies have shown the positive effect of blue light on chlorophyll content [46,47,48,49].

We found that for LED lighting, red and green channels overlap better (see Figure 6, second column). As a result, yellow light appears, indicating better colocalization of chlorophylls and carotenoids. In addition, the chlorophyll distribution (red channel) is more evenly balanced within chloroplasts grown under LED lighting. Changes in the distribution of chlorophylls and carotenoids under HPS or LED supplementary lighting may reflect adaptation of the photosynthetic apparatus toward different light spectra. These phenomena require further investigation, as the greenhouse environment may reduce the effects of specific spectra on the distribution of the photosynthetic pigments within chloroplasts.

## 3. Materials and Methods

### 3.1. Plant Materials and Growth Conditions

This study was conducted in a greenhouse located 21° E, 51°15′ N during the winter cycles of 2018 and 2020. The cultivation of bell pepper cultivars with fleshy, block-type, and red fruit is of the greatest importance in the cultivation of peppers under covers, although cultivars with very sweet and elongated fruit are also becoming increasingly important. Two red-fruited, sweet pepper cultivars were studied, namely ‘Aifos F1′ (Bayer Crop Science—Seminis), with block-type fruit and soft flesh, and ‘Palermo F1′ (Rijk Zwaan), with elongated fruit and thinner flesh. Two-week-old pepper seedlings were planted into rockwool cubes on 5 December in both years. Half of the plants were supplemented with light from HPS lamps (Gavita GAN 400 W Figure S1 in the Supplementary Materials) and the other half with Green Power LED lamps (DR/W–LB, 195 W, Figure S2 in the Supplementary Materials) (Philips). The LED lamp characteristics were as follows: 87.5% red light in the wavelength range of 630 to 660 nm, with the highest proportion at 660 nm, and 12.5% blue light in the range of 440 to 460 nm. The daily light exposure equalled 16 h. In the experimental greenhouse where pepper seedlings were grown, the PAR (photosynthetically active radiation) light level was ~170 μmol m^−2^ s^−1^ (PPFD—photosynthetic photon flux density). The lighting lamps switched off automatically when the solar radiation was higher than 250 W m^−2^. The average daily solar radiation during the seedling growth period was around 186.9 J cm^−2^. In the growing compartment of the greenhouse, the temperature was maintained at 22 °C during the day and 20 °C at night, with RH kept over a range of approximately 60–70% and CO_2_ at an average concentration of 800 ppm. The nutrient solution for the supply of seedlings contained the following components in mg·dm^−3^: N–NO_3_-195, P-57, K-273, Mg-47, Ca-187, Fe-2, Mn-0.6, B-0.3, Cu-0.15, Zn-0.3, Mo-0.05. The average pH and EC were respectively about 5.5 and 2.8 dS m^−1^. The study adopted a completely randomized design. Equally sized plots containing 10 plants of each cultivar were drawn in the experimental compartment, with three replications of 10 plants in each combination. The test material consisted of 120 plants, each in identical rockwool cubes.

### 3.2. Morphological Characteristics

Measurements of plant height and leaf number per plant were taken weekly on five randomly selected plants from each repetition, after 7, 14, 21, and 28 days of cultivation. The plant height and leaf number results are given for each plant based on averages from the measurements taken.

### 3.3. Chlorophyll Fluorescence Analysis

Every week, the relative contents of chlorophyll a + b in the fully mature leaves were measured (using a Minolta SPAD-502 apparatus). Measurements were taken from 5 randomly selected plants from the combinations. The results were averaged from the five-unit measurements taken from the examined leaves. The chosen chlorophyll fluorescence was measured under ambient light and after dark adaptation with special leaf clips on the same leaves (spots) of pepper plants using the following equipment:

- FMS-2 Field-Portable Pulse-Modulated Chlorophyll Fluorescence Monitoring System (Hansatech Instruments Ltd., King’s Lynn, Norfolk, England). Measurements were taken under ambient light. The saturating light source used was a built-in halogen lamp. The pulse intensity was equal to 8000 μmol·m^−2^ s^−1^ and the pulse duration was 1 s. The following parameters were measured: Fs—steady-state fluorescence yield; Fm’—light-adapted fluorescence maximum; ΦPSII—quantum efficiency of PSII;
- A Pocket PEA fluorimeter (Hansatech Instruments Ltd., King’s Lynn, Norfolk, England) was used to measure prompt fluorescence, obtaining measurements of the maximum efficiency of the plant’s photosynthetic rate after 30 min of dark adaption of the leaves (using special clips). The following parameters were analyzed, where Fv/Fm is the maximum efficiency of the PSII photochemistry (Fv/Fm) and the performance index (PI) is displayed as PI_Abs_ or PI_Ins_. The latter is calculated on the basis of the following formula:
  (1) $${\mathrm{PI}}_{\mathrm{ABS}}=\frac{\mathrm{RC}}{\mathrm{ABS}}\cdot \frac{\phi_{\mathrm{po}}}{1-\phi_{\mathrm{po}}}\cdot \frac{\psi_{o}}{1-\psi_{o}}$$
  where RC is the reaction center; ABS is the absorption; ϕ_po_ = F_V_/F_M_, which is the maximum quantum yield of the primary photochemical reactions (at *t* = 0), which proves the probability of trapping the energy of absorbed photons (i.e., excitons migrating along an antenna) by PSII reaction centers; ψ_o_ is the probability (at *t* = 0) of electron transport outside Q_A_^−^, i.e., that an RC trapped exciton moves an electron into the electron transport chain outside Q_A_^−^.

### 3.4. Light and Transmission Electron Microscope

For the anatomical observations, after the 28th day of cultivation, ten fragments from 10 fully expanded leaves (counting from the tip) collected from 3 seedlings of pepper from two cultivars grown in the greenhouses under different lighting systems were fixed for 3 h in Karnovsky [50] medium, then afterwards treated with 1% osmium tetraoxide for 3 h, dehydrated in an ethanol series containing propylene oxide and embedded in epoxy resin (Serva, Heidelberg, Germany). After being embedded in resin, leaf samples were then cut on a Jung microtome (RM 2065) into semithin sections (3 µm) and stained with 1% toluidine blue prior to examination under a light microscope (Olympus-Provis). Taking five leaves for each ecotype, the total thickness, number, thicknesses of the palisade and spongy cell layers, sizes of the parenchyma and spongy cells, and stomata number per 600 μm of leaf surface were measured with the Olympus-Provis cellSens Standard program at 10× magnification. Statistical analysis was performed in STATGRAPHICS Plus 5.1. For parametric tests, ANOVA was used. For chloroplast analysis, ultra-thin (80 nm thick) leaf sections were taken from epoxy-resin-embedded samples using a Leica UCT ultramicrotome (Leica Microsystems, Nussloch, Germany) and collected on formvar-coated grids, which were short-stained with uranyl acetate and lead citrate and examined under an FEI 268D ‘Morgagni’ (FEI Comp., Hillsboro, OR, USA) transmission electron microscope (TEM) equipped with a 10 MPix Olympus-SIS ‘Morada’ digital camera (Olympus-SIS, Münster, Germany). Digital images were saved as jpg files and were adjusted using Photoshop CS 8.0 (Adobe Systems, San Francisco, CA, USA) software using non-destructive tools (contrast or levels) if necessary.

### 3.5. Confocal Laser Scanning Microscope

A confocal laser scanning microscope (Leica TCS SP5II, Leica Microsystems CMS, Wetzlar, Germany) was used to examine groups of chloroplasts located in palisade cells of the mesophyll of pepper leaves. Fluorescence imaging of chlorophyll and carotenoids was performed on freshly prepared cross-sections through the leaf, which were prepared using razor blades. Tiny slices were embedded in distilled water. Carotenoid and chlorophyll fluorescence was excited at 488/633 nm and recorded at 520–580/660–705 nm at room temperature. Sequential scanning was performed in order to avoid bleed-through fluorescent signals between channels. In total, 29 images were acquired at intervals of 0.18 µm along the *z*-axis. All image series were deconvoluted to remove background noise and improve image quality. To show the distribution of the chlorophyll and carotenoids, red and green colors were digitally separated. The intensity levels of chlorophyll and carotenoid fluorescence were recorded separately in the central parts (the region of interest was a circle measuring 1 µm in dimeter) of chloroplasts while avoiding the starch granules.

### 3.6. Statistical Analysis

Statistical analysis was performed using two-way analysis of variance (ANOVA). Detailed comparison of the means was performed using Tukey’s test at a significance level of α = 0.05.

## 4. Conclusions

Regarding pepper seedling growth, our study revealed that LEDs delivered a better blue-to-red (R/B) ratio than HPS lamps. This better supplemental lighting (LEDs) indirectly enhanced plant growth by increasing the height of the seedlings and the number of leaves; however, we found that this effect is cultivar-dependent.

We believe that behind the above-mentioned growth enhancement were many anatomical and physiological changes that resulted in better photosynthetic performance and biomass production, such as thicker leaves, longer palisade parenchyma cells, larger mesophyll cells, increased chloroplast and chlorophyll contents, and better photosynthetic efficiency.

We recommend the use of the performance index (PI) parameter to monitor the physiological statuses of plants while testing new supplemental lighting. Further studies should investigate the responses of plants to sunlight alone and also to a combination of sunlight and different artificial light sources with different R/B ratios.

## Figures and Tables

**Table 1:** Effects of supplementary lighting with sodium and LED lamps on the photosynthetic activity of pepper seedling leaves according to the cultivar (averages of four measurement dates).

Chlorophyll Fluorescence Parameters	Cultivar	HPS	LED
φPSII	‘Aifos’	0.78 a	0.74 bc
	‘Palermo’	0.77 ab	0.72 c
	Average	0.77 A	0.73 B
Fv/Fm	‘Aifos’	0.83 a	0.81 b
	‘Palermo’	0.82 ab	0.80 c
	Average	0.82 A	0.80 B
PI inst.	‘Aifos’	4.41 bc	6.41 a (45%)
	‘Palermo’	3.81 c	4.97 b (30%)
	Average	4.11 B	5.70 A (38.7%)
DIo/RC	‘Aifos’	0.21 ab	0.19 b
	‘Palermo’	0.23 a	0.23 a
	Average	0.22 A	0.20 A
Area	‘Aifos’	773,108 ab	814,715 a
	‘Palermo’	725,554 ab	697,771 b
	Average	749,331 A	756,243 A

The mean values marked with the same letters do not differ significantly according to the Tukey HSD test at α = 0.05. The small letters indicate the differences in the interaction of cultivar x lamp type, the capital letters indicate the differences in the lamp type.

**Table 2:** Analyzed leaf traits depending on the type of supplementary lighting and cultivar.

Analyzed Traits	‘Aifos’ HPS	‘Palermo’ HPS	‘Aifos’ LED	‘Palermo’ LED
Total thickness (µm)	195.09 ± 13.4 b	193.05 ± 12.0 b	264.05 ± 8.9 a	276.74 ± 13.2 a
Number of palisade cell layers	1	1	1	1
Length of palisade parenchyma cells (µm)	59.82 ± 6.3 b	63.44 ± 7.2 b	91.18 ± 5.2 a	109.14 ± 11.1 a
Width of palisade parenchyma cells (µm)	27 ± 0.1 b	22.79 ± 0.4 b	34.25 ± 12.2 b	75.03 ± 19.2 a
Number of spongy cell layers	3	3	3–4	3–4
Thickness of spongy layer (µm)	129.02 ± 6.2 b	122.5 ± 4.3 b	180.02 ± 12.4 a	160.6 ± 2.6 a
Length of spongy parenchyma cells (µm)	65.25 ± 5.5 b	73.77 ± 8 ab	40.45 ± 25.0 b	103.66 ± 3.2 a
Width of spongy parenchyma cells (µm)	31.03 ± 5.2 b	39.23 ± 8.36 b	57.61 ± 4.7 a	36.64 ± 6.3 b
Stomata number in adaxial epidermis	2 ± 0.5 a	0 ± 0.0 b	3 ± 0.6 a	3 ± 0.6 a
Stomata number in abaxial epidermis	4 ± 0.6 b	4 ± 0.6 b	4 ± 0.0 b	6 ± 1.1 a

Mean values marked with the same letters are not significantly different according to Tukey’s HSD test at α = 0.05. Values are means ± SD of 3 plants per treatment.

## Data Availability

Data are available from the authors upon request.

## Supplementary Materials

The following are available online at www.mdpi.com/10.3390/plants1340667/s1, Figure S1: The spectrum of HPS lamps measured during cultivation with the Gigahertz-Optik apparatus, Figure S2: The spectrum of LED lamps measured during cultivation with the Gigahertz-Optik apparatus.
