# Supplementary material for: Photosynthetic Efficiency and Anatomical Structure of Pepper Leaf (Capsicum annuum L.) Transplants Grown under High-Pressure Sodium (HPS) and Light-Emitting Diode (LED) Supplementary Lighting Systems
Source: Plants (Basel). 2021 Sep 22;10(10):1975. doi: 10.3390/plants10101975 (PMC8541379; doi:10.3390/plants10101975)
Supplement: Supplementary file 1 [file plants-10-01975-s001.zip › supplementary.v2.pdf]

Supplementary to :

Photosynthetic Efficiency and Anatomical Structure of Pepper Leaf (*Capsicum annuum* L.) Transplants Grown under High-Pressure Sodium (HPS) and Light-Emitting Diode (LED) Supplementary Lighting Systems

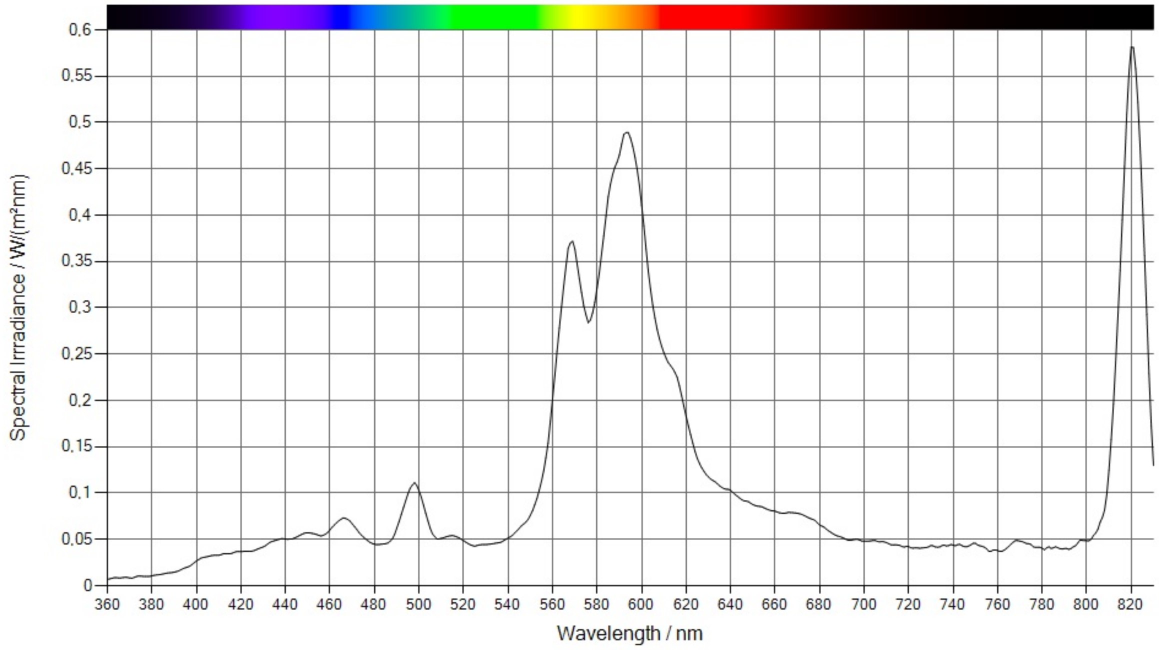

Supplementary Figure S1. The spectrum of HPS lamps measured during cultivation with the Gigahertz-Optik apparatus

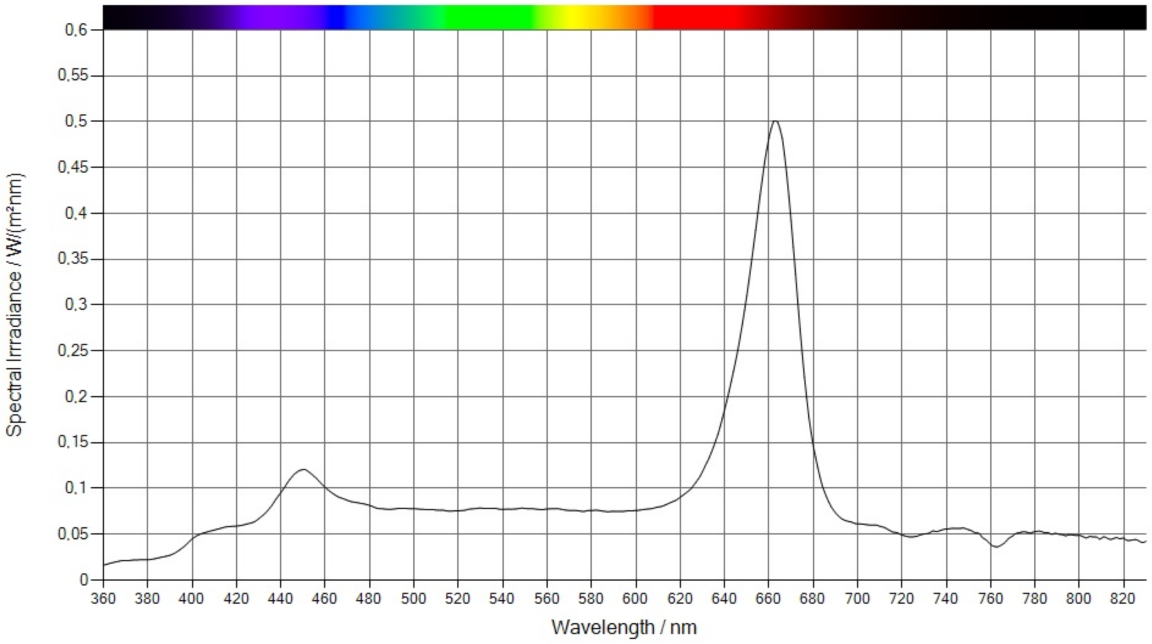

Supplementary Figure S2. The spectrum of LED lamps measured during cultivation with the Gigahertz-Optik apparatus
